# Antioxidant and Biological Activities of the Lotus Root Polysaccharide-Iron (III) Complex

**DOI:** 10.3390/molecules27207106

**Published:** 2022-10-21

**Authors:** Shuai Yuan, Pei-Yu Dong, Hao-Hai Ma, Sheng-Lin Liang, Long Li, Xi-Feng Zhang

**Affiliations:** College of Veterinary Medicine, Qingdao Agricultural University, Qingdao 266109, China

**Keywords:** lotus root, polysaccharide, polysaccharide iron, antioxidant, metabolome, microbiota

## Abstract

In this study, the synthesis parameters of the lotus root polysaccharide iron complex (LRPF) were determined and optimized by response surface methodology. Under the optimum preparation conditions, the pH of the solution was 9, the ratio of M (trisodium citrate): m (lotus root polysaccharide) was 0.45, the reaction time was 3 h. UV spectroscopy, thermogravimetry, FT-IR spectroscopy, X-ray diffraction, CD, and NMR were used for the characterization of the LRPF. LRPF has good stability and easily releases iron ions under artificial gastrointestinal conditions. LRPF exhibited antioxidant activity in vitro and can significantly improve the antioxidant activity in vivo. In addition, LRPF has a good effect in the treatment of iron deficiency anemia in model mice, impacts the gut microbiome, and reduces the iron deficiency-induced perniciousness by regulating steroid hormone biosynthesis. Therefore, LRPF can be used as a nutritional supplement to treat and prevent iron-deficiency anemia and improve human immunity.

## 1. Introduction

*Nelumbo nucifera Gaertn* is a large perennial herb in the water lily family. *Nelumbo nucifera Gaertn* is native to China with a long planting history and India but is widely planted in Southeast Asia. It tastes slightly crisp and sweet and is an aquatic cash crop for medicine and food [1,2]. According to the records in ancient medical books, *Lotus root* has many effects, such as eliminating inflammation, relieving thirst, and alleviating diarrhea [3]. The current research found that lotus root contains a variety of proteins, minerals, polysaccharides, vitamins, and other active components, which have high medicinal value. *Lotus root* polysaccharide is one of the main active components of *Lotus root*, which has many effects, such as anti-oxidation, reducing blood lipids, regulating immune activity, and bacteriostasis [4,5,6]. In recent years, plant polysaccharides have been widely concerned because of their high efficiency, rich and diverse biological activities, and low toxicity [7].

Iron is an essential trace element in the human body. It mainly participates in the transportation and storage of oxygen in blood in the form of hemoglobin and myoglobin and plays a vital role in regulating tissue respiration [8]. Iron deficiency and iron deficiency anemia are among the leading causes of malnutrition worldwide, and it is also the most urgent problem of micronutrient deficiency. Iron deficiency anemia has long been treated with the regular use of iron supplements, such as ferrous sulfate. However, the side effects of oral iron supplements should not be ignored, with 30 to 50 percent of patients reporting adverse effects after taking them. Most polysaccharides can react with ferric iron to synthesize the polysaccharide iron complex [9]. It is a stable complex with a ferric core as the center and polysaccharide as the ligand, which is connected with iron through the O bridge and OH bridges. Polysaccharide iron complex not only has stable structural properties, does not contain free iron ions, and has no side effects on the intestine and stomach, but also retains the biological activity of polysaccharide itself in addition to treating and preventing iron-deficiency anemia [10,11].

Although polysaccharide iron complex has been recognized as a potential drug for the treatment of iron-deficiency anemia, the effects of polysaccharide iron complex on the treatment of anemia symptoms and the effects on organisms are still unclear. In this study, response surface methodology (RSM) was used to study the preparation of LRPF, and the antioxidant effect of the *Lotus root* polysaccharide iron complex was also studied. Iron-deficiency anemia mice were used as a model animal to investigate the biological activities of this compound.

## 2. Results and Discussion

### 2.1. Optimization of Synthesis Parameters of LRPF

According to the design principle of the Box –Behnken center combination test, response surface analysis (RSM) with three factors and three levels was carried out to optimize the synthesis parameters. The effects of reaction pH (X_1_), mass ratio of trisodium citrate to LRP (X_2_) and reaction time (X_3_) on the iron content of LRPF were studied (Supplemental Table S1). The design was optimized through the Design-Expert 8.0 software process. The parameters and results of the 17 combinations were designed as random sequential tests based on iron content, and the experimental results are listed in Supplemental Table S2. The predicted response result (y) of the iron content of LRPF can be fitted to the following polynomial equation:

Y = −31.04587 + 6.67187X_1_ + 1.66275X_2_ − 0.87650X_3_ − 0.013250X_1_X_2_ + 0.46200X_1_X_3_ + 0.22500X_2_X_3_ − 0.38075X_1_^2^ − 0.27600X_2_^2^ − 0.42800X_3_^2^

Supplemental Table S3 shows the results of the analysis of variance (ANOVA) of the response surface quadratic model. The *p*-value is used to represent the significance of each factor, and the F value is used to reflect the statistical significance of each factor in the regression equation [12]. It can be seen that X_2_, X_1_X_2_, X_1_^2^, X_2_^2^, and X_3_^2^ have highly significant effects, indicating that the above process parameters are highly correlated with the iron content of lotus root polysynthesis. The model correction judgment coefficient R^2^_adj_ = 0.9732 shows that the model can predict 97.32% of the response results, which shows that the model can accurately predict the experimental results. The judgment coefficient R^2^ = 0.9883 is close to R^2^_pred_ = 0.9278, indicating that the model has an excellent fitting degree, the stress surface regression equation is reliable, and there is no need for further optimization.

Through response surface analysis, the optimal synthesis conditions of LRPF were predicted by regression equation and data model (X_1_ = 9, X_2_ = 0.45, X_3_ = 3). The reliability of the model was verified by three parallel experiments under optimal conditions. The average of the actual results was 1.1383. Compared with the theoretical value of 1.19083, the coincidence rate is 95.66%, and the relative error is 4.34%. The experimental results show that the response surface model is accurate and reliable.

### 2.2. Response Surface Diagram with Contour

**Figure 1:**
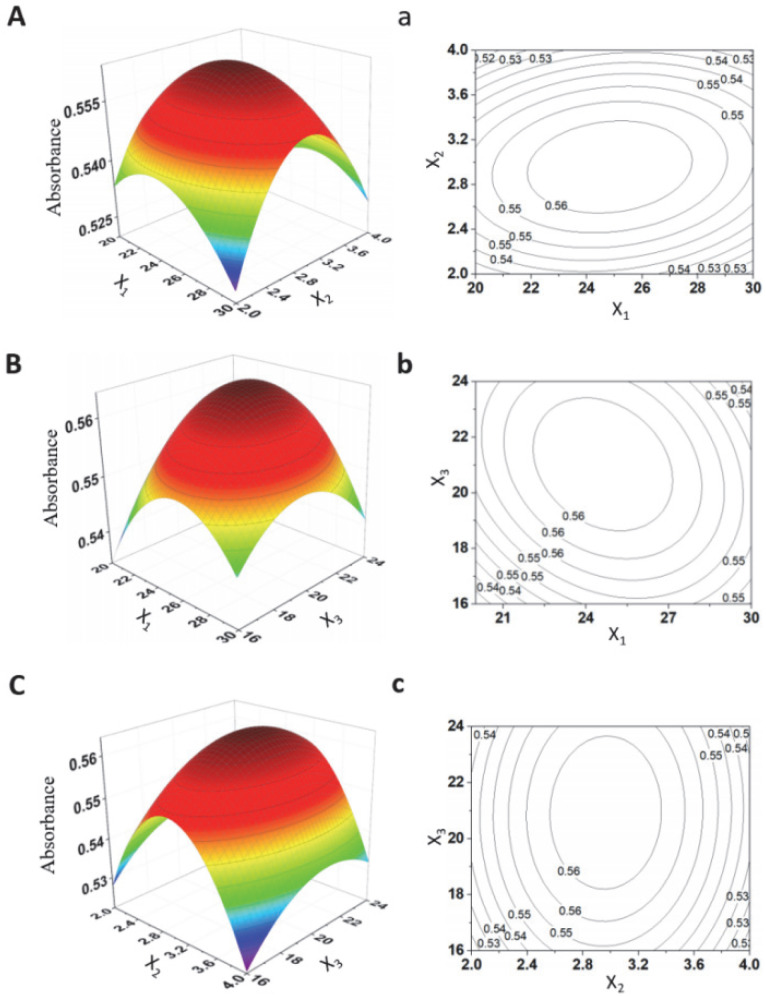
Response surface plots showing effects of variables on the iron content of LRPF. (**A**,**a**) Plots of the effect of reaction pH, mass ratio of trisodium citrate to LRP and their interaction. (**B**,**b**) Plots of the effect of reaction pH, reaction time and their interaction. (**C**,**c**) Plots of the effect of mass ratio of trisodium citrate to LRP, reaction time and their interaction.

The Design-Expert software was used to draw how the parameters and their interaction affect the yield of iron content. The iron content of LRPF is affected by pH, the mass ratio of trisodium citrate to lotus root polysaccharide, and reaction temperature. The results are shown in the Figure 1. According to the regression equation, draw a three-dimensional surface diagram. The top and its vicinity of the surface represent the range of the best response value under the interaction of each experimental factor. The inclination of the surface determines the influence of the two interaction parameters on the prediction results. That is, the surface is steeper, which indicates that the interaction of the two factors has a more significant influence on the prediction results. The contour shape of the contour map reflects the correlation between the two interactive factors. If the contour shape is oval, the interaction between the two factors is significant. In Figure 1, it can be seen from the three-dimensional spatial surface diagram that the spatial surface curvature of the reaction time and reaction pH is the largest, and that it has the most significant effect on the iron content of the product. In contrast, the effect of M (trisodium citrate): m (polysaccharide) is slightly weak. The contour plot results also show that the contour shape of the reaction time and reaction pH is circular, and their interaction is the strongest.

### 2.3. Spectroscopic Characterization of the LRPF

#### 2.3.1. UV Analysis

**Figure 2:**
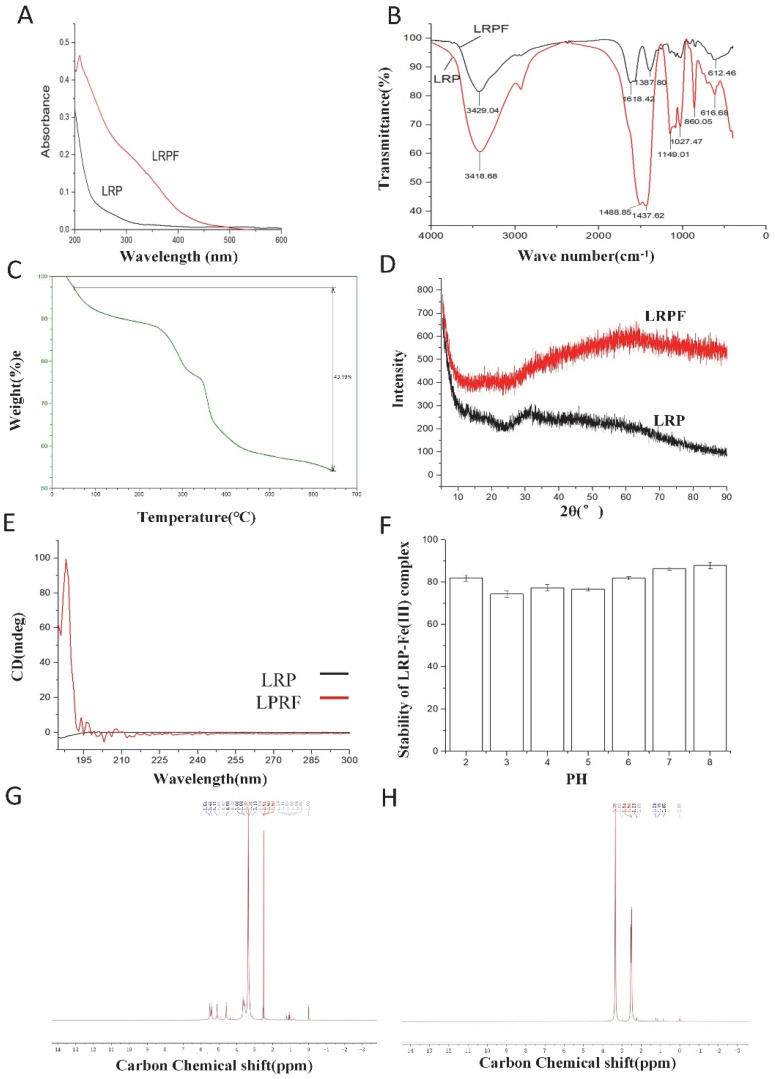
Characterization of LRP and LRPF. (**A**) Ultraviolet spectroscopy of LRP and LRPF. (**B**) FT-IR spectrum of LRP and LRPF. (**C**) Thermogravimetric spectrum of LRPF. (**D**) X-ray diffraction spectrum of LRP and LRPF. (**E**) Circular dichroism spectrum of LRP and LRPF. (**F**) The stability of LRPF in vitro. (**G**) ^1^H NMR spectra of LRP. (**H**) ^1^H NMR spectra of LRPF.

The UV spectra of LRP and LRPF (Figure 2A) show that the UV spectrum curve of LRP in the wavelength range of 200–600 nm is relatively smooth. Compared with LRP, LRPF has an apparent absorption peak at 210 nm, indicating that the structure of LRP has changed significantly after synthesis [13]. The UV absorption curve of LRPF at 300–400 nm is because the binding of LRP with iron ions will increase its own UV absorption intensity, indicating the successful construction of LRPF.

#### 2.3.2. FT-IR Analysis

The infrared spectrum scanning of LRP and LRPF (Figure 2B) shows that both LRP and LRPF have typical polysaccharide characteristic absorption peaks, and the complexation of iron ions will not destroy the original structure of the polysaccharide [14]. The absorption peak at 3400 cm^−1^ wavelength is O-H stretching vibration [13], the absorption peak at 2900 cm^−1^ wavelength is C-H stretching vibration [15], the absorption peak at 1600 cm^−1^ is C=O vibration, and 1500 cm^−1^ may be relative to vibration of -C=C- [16]. These results suggest that free carboxyl groups and uronic acids may exist in the samples [17]. In addition, the absorption peaks of lotus root polysaccharide and lotus root polysaccharide iron at the wavelength of 1400 cm^−1^, 1150 cm^−1^, 1080 cm^−1^, 1025 cm^−1^ are mainly due to the existence of a pyran ring structure, resulting in the stretching of C-O and the bending vibration of C-O-H [18]. The absorption peak of iron in lotus root polysaccharides at 860 cm^−1^, 612 cm^−1^ wavelength is the characteristic absorption peak of the β-FeOOH structure [19], indicating that iron has been successfully synthesized into lotus root polysaccharides. The absorption peak pattern of LRP and LRPF in the wavelength range of 500–1200 cm^−1^ and the change of peak height may be caused by the ion polarization caused by the complexation of iron to polysaccharide molecules, indicating that LRPF also has the active group of LRP based on successful synthesis.

#### 2.3.3. TG Analysis

A thermogravimetric analysis (TG) curve reflects the relationship between sample weight and temperature. The thermogravimetric analysis results (Figure 2C) of iron in the LRP-Iron Complex are shown in the Figure. When the temperature reached 100 °C, the mass of the LRP-Iron Complex remained at 92%, and the heat loss rate of LRPF was stable between 100 °C and 280 °C. When the temperature increased from 280 to 360 °C, the LRPF decomposed violently. When the temperature reached 650 °C, the residual mass of LRPF was 56.81%. Thermogravimetric analysis results showed that LRPF had good thermal stability in the temperature range of 50–650 °C.

#### 2.3.4. XRD Analysis

The X-ray diffraction results of LRP and LRPF are shown in the Figure. In Figure 2D, it can be seen that LRP has a major sugar chain characteristic peak near 32° [20]. No characteristic diffraction peaks of LRPF are observed because of its loading content and weak crystallization, on the other hand, also implying the good dispersion of the very small iron on the LRP. In addition, the signal value of LRPF is significantly enhanced due to the successful synthesis of iron ions into the polysaccharide molecular chain, which improves the crystal strength of LRPF.

#### 2.3.5. CD Analysis

The signal curve of LRP was relatively flat (Figure 2E), indicating that the molecular morphology of LRP in an aqueous solution was fairly symmetrical, and there was no irregular configuration [21]. After the complexation of LRP with iron ions, significant absorption peaks appeared at 192 nm and 194 nm, which is contributed to the carboxyl group of metal ions causing the polysaccharide chains to polymerize one another to form an “egg-box” structure, resulting in the change of the original molecular configuration of polysaccharide [22].

#### 2.3.6. ^1^H NMR Analysis

The NMR results of LRP and LRPF are shown in Figure 2. There are multiple characteristic NMR absorption peaks of LRP in the range of displacement of 4.0–5.5 ppm, and its hydrocephalic proton absorption peaks are concentrated in the range of displacement of 5.5–6.0 ppm (Figure 2G) [23]. The NMR hydrogen spectrum of the LRPF showed not many other signal peaks except for the H-2 signal on the glucuronic acid residue at a shift of 3.3 ppm (Figure 2H). As is due to the spin of iron in the magnetic field will form a specific magnetic field blind area, weakening the original polysaccharide characteristic peak, resulting in invisibility on the nuclear magnetic spectrum [20]. In our study, the presence of the “blind zone,” i.e., the disappearance of absorption peaks caused by the complexation between LRP and iron (III), suggests that the LRP is bonded to iron (III) in the LRPF. These results further indicate that the LRPF could be potentially used as an effective chelator of iron (III) for a novel human oral iron supplement.

### 2.4. Antioxidant Activity of LRPF

#### 2.4.1. The ABTS Radical Scavenging Rate

**Figure 3:**
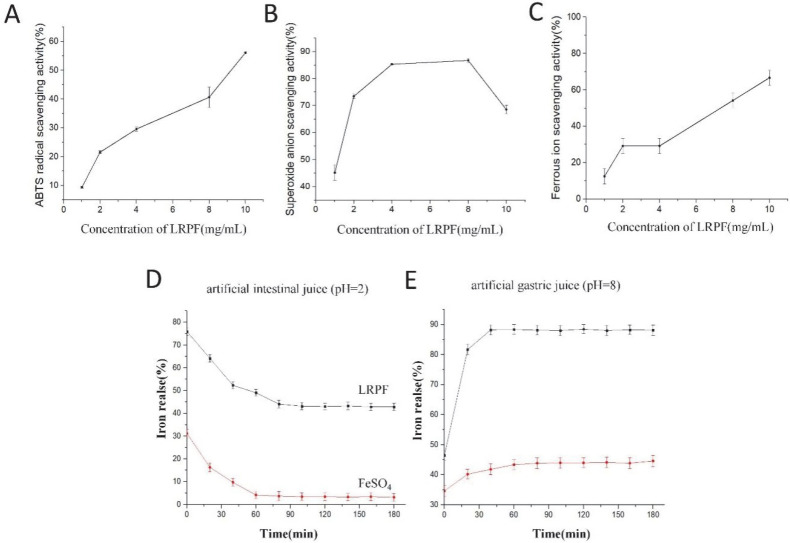
Antioxidant activity of LRPF and Iron Release In Vitro Assay of LRPF and FeSO_4_. (**A**) The ABTS radical scavenging activity of LRPF and FeSO_4_. (**B**) Metal ion scavenging activity of LRPF and FeSO_4_. (**C**) Superoxide-radical scavenging activity of LRPF and FeSO_4_. (**D**) The iron release of LRPF and FeSO_4_ in artificial intestinal juice (pH = 2). (**E**) The iron release of LRPF and FeSO_4_ in artificial gastric juice (pH = 8).

ABTS can react with K_2_S_2_O_8_ to form stable cationic free radicals, and antioxidants can effectively inhibit the oxidation reaction, weakening its UV absorption peak at 734 nm. Therefore, it is used as one of the indicators to measure antioxidant activity [24]. The ABTS radical scavenging activity of LRPF (Figure 3A) is showing an evident concentration-dependent trend. When the concentration reached 10 mg/mL, the ABTS radical scavenging activity peaked, and the ABTS radical scavenging activity was 56.08%.

#### 2.4.2. Superoxide-Radical Scavenging Activity

The superoxide-radical scavenging activity of LRPF (Figure 3B) showed a trend of first increasing and then decreasing with the increase of its own concentration. When the concentration increases from 2 mg/mL to 8 mg/mL, the superoxide-radical ion activity gradually increases, up to 86.71%, which may be due to the high concentration inhibition caused by the high concentration of LRPF.

#### 2.4.3. Metal Ion Scavenging Activity

The antioxidant activity of LRPF through metal ion scavenging activity is shown in Figure 3C. The ferrous ion scavenging ability of LRPF was increased with the increased concentration of LRPF, and its maximum scavenging ability was 66.67% at the concentration of 10 mg/mL.

### 2.5. Physicochemical Characterization of the LRPF

#### 2.5.1. The Iron Release of LRPF In Vitro

The release of Fe refers to the fact that we studied the dissolution of Fe ions in LRPF in an environment with pH resemblance to human gastrointestinal digestion. Figure 3D,E shows the changes in the iron release of LRPF and FeSO_4_ in artificial intestinal fluid and human gastric juice. The release of LRPF and FeSO_4_ in gastric juice was low initially, but the iron release of LRPF increased rapidly with time. After digestion for 0.5 h, the iron release reached 85.32%, while the iron release of FeSO_4_ did not exceed 45%. Under the condition of alkaline intestinal fluid, insoluble iron compounds may be formed in the alkaline environment of the intestinal medium and cannot be absorbed by the small intestine [25]. With the extension of digestion time, the iron release of LRPF and FeSO_4_ decreased. When the digestion time reached 3 h, the iron release of LRPF was stable at 40%, while the iron release of FeSO_4_ was less than 10%. It can be explained that compared with FeSO_4_, LRPF can maintain better solubility and stability in the gastrointestinal tract in vitro and can be well absorbed in the subsequent digestion process.

#### 2.5.2. The Stability of LRPF In Vitro

The value of the bar graph in Figure 2F indicates the residual iron content of LRPF in the solution after a certain amount of time. The higher the residual iron content is, the more stable the sample is under this condition. Error bars indicate the degree of deviation of the data across multiple experiments.

LRPF has shown strong stability in neutral and weakly alkaline environments in Figure 2F. After 4 h, it can still retain more than 80% iron content. In addition, the stability of LRPF decreased slightly in an acidic environment due to the degradation of polysaccharide by low pH values. Still, the overall stability was more than 75%, which proved that LRPF is structurally stable in vitro at different pH values.

### 2.6. Antianemia Function Test of the LRPF

**Figure 4:**
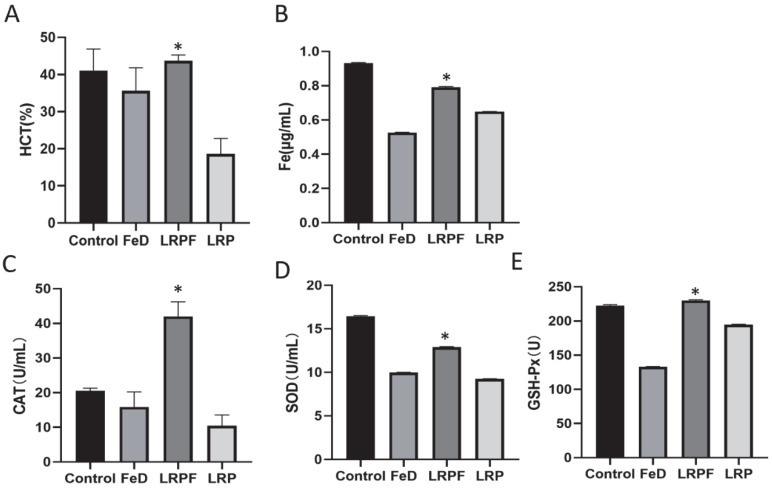
Effects of LRPF on blood parameters and antioxidant indexes in the model mouse of iron deficiency anemia. The level of HCT, Fe, CAT, MDA, SOD and GSH-PH in serum. (**A**) HCT. (**B**) Fe. (**C**) CAT. (**D**) SOD. (**E**) GSH-Px. (* *p* < 0.05).

The results of the antianemia test show that LRPF can effectively treat iron deficiency anemia (Figure 4 and Supplemental Figure S1). The RBS (Red Blood Cell), hemoglobin concentration (HGB), mean red cell volume (MCV), hematocrit (HCT), mean corpuscular hemoglobin (MCH) and platelet count (PLT) of iron deficiency group mice (FeD) and LRP group mice were lower than the control group. The two groups of mice were in a state of iron-deficiency anemia. After four weeks of treatment with LRPF, their blood parameters were entirely or partially repaired, and the physiological states of mice were improved. These data clearly show that LRP-Iron Complex significantly improves various blood parameters of iron-deficiency anemia mice to improve iron deficiency. LRPF has great potential and application value in treating iron deficiency anemia and as an iron supplement. As shown in Figure 4B, compared with the control group, the serum iron content of the iron deficiency (FeD) group (0.53 μg/mL) was significantly lower, which accorded with the characteristics of iron deficiency anemia. The serum iron content in the blood of LRP-treated mice was still lower than that in a blank group, only 0.65 μg/mL, which indicated the LRP itself did not have any iron supplement effect. The serum iron content of mice in the LRPF group increased to 0.79 μg/mL. It was shown that LRPF could effectively supplement iron and relieve the symptoms of low iron content in the blood caused by iron deficiency anemia (* *p* < 0.05).

Previous experimental data showed that LRPF had an excellent antioxidant activity in vitro. Therefore, we measured CAT, SOD, GSH-Px, and TNF-α in mouse serum. The results are shown in Figure 4 and Supplemental Figure S1. From the results, it can be seen that, compared with the FeD group, the levels of CAT, SOD, and GSH-Px in the LRPF group were increased significantly, and TNF-α decreased significantly (Supplemental Figure S1I). Compared with the FeD group, white blood cell count (WBC) and lymphocyte count (LYM) in the LRPF group were softened by subsidized iron treatment (Supplemental Figure S1F,M). Taking LRPF can not only effectively prevent and treat iron deficiency anemia, but also significantly improve the body’s antioxidant capacity and cause inflammation (Supplemental Figure S1I).

### 2.7. The Influence of LRPF on Iron Deficiency-Induced Gut Dysfunction

**Figure 5:**
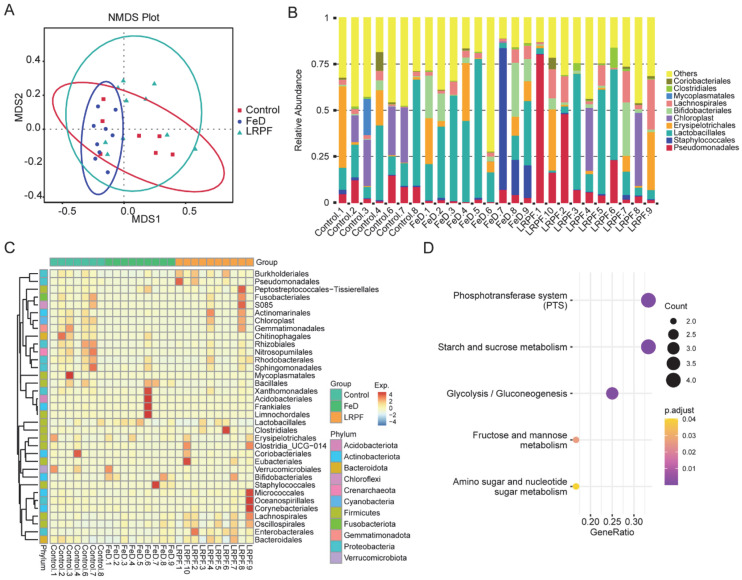
Effects of LRPF on the intestinal flora of mice with iron-deficiency anemia. (**A**) Nonmetric multidimensional scaling (NMDS) plot showing the relationship between samples. The red square means the control group, the blue dot means the iron deficiency (FeD) group, and the green triangle means the LRPF group. (**B**) The relative abundance of the top 10 microbiotas at the order level. Different colors mean different microbiotas. (**C**) The heat map showing the top 35 microbiotas at the order level. (**D**) The dot plot showing the top 5 KEGG signaling pathways of the top microbiotas.

To examine the role of LRPF on the gut microbiota, the bacterial 16s rRNA V3-4 region of the gut chyme was sequenced. Non-nitric multidimensional scaling (NMDS) analysis showed data to be reliable for further research (Figure 5A). The top 10 microflora at order level was obtained, and we found that LRPF significantly increased the *Pseudomonadales* (Figure 5B). Further, the heat map of relative abundance of the top 35 microflora suggested that after iron deficiency, *Frankiales, Limnochordales, Xanthomonadales*, and *Acidobacteriales* were increased. LRPF treatment decreased *Frankiales, Limnochordales, Xanthomonadales* and *Acidobacteriales,* and improved the expression of *Microcooccales, Oceanospirillales, Corynebacteriales* (Figure 5C). Interestingly, the functional enrichment analysis of gut microbiota showed that the microbiotas worked by regulating the phosphotransferase system, starch, and sucrose metabolism (Figure 5D).

### 2.8. The Influence of LRPF on Blood Metabolites

**Figure 6:**
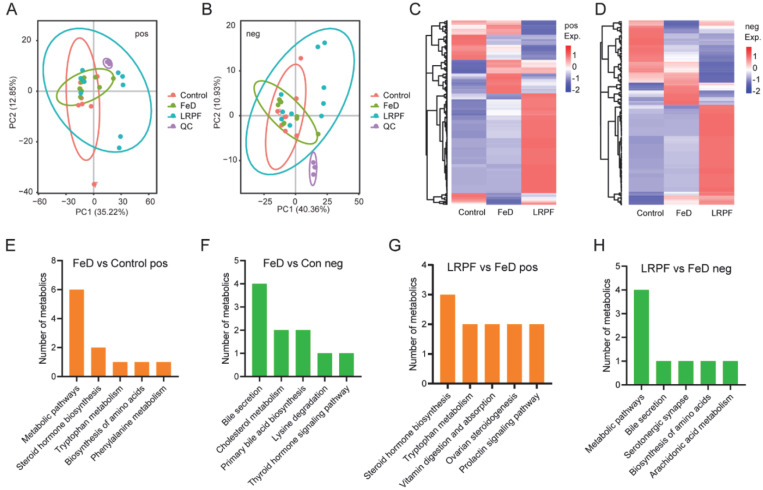
Effects of LRPF on the metabolome of mice with iron deficiency anemia. (**A**) The dot plot showing the top 2 PCs of positive ion (pos) metabolite. (**B**) The dot plot showing the top 2 PCs of negative ion (neg) metabolite. (**C**) The heat map showing the expression trends of the pos metabolites. (**D**) The heat map showing the expression trends of the neg metabolites. (**E**) The top 5 KEGG signaling pathways of differentially expressed pos metabolites between FeD vs. the control group. (**F**) The top 5 KEGG signaling pathways of differentially expressed neg metabolites between FeD vs. the control group. (**G**) The top 5 KEGG signaling pathways of differentially expressed pos metabolites between LRPF vs. the FeD group. (**H**) The top 5 KEGG signaling pathways of differentially expressed neg metabolites between LRPF vs. the FeD group.

After discovering that LRPF can affect the gut microbiome, we next began to explore whether LRPF could affect blood metabolism. We performed PCA analysis to test the consistency of the sample, and the results showed that there were significant differences in blood metabolites between the different groups, especially the LRPF group (Figure 6A,B). Further differential analysis revealed that most of the metabolites in blood were upregulated after LRPF treatment (Figure 6C,D). Once we obtained differential metabolites in different groups, we attempted to explore the functions of these metabolites. The KEGG analysis showed that iron deficiency mainly impaired metabolic pathways and steroid hormone biosynthesis (Figure 6E). Moreover, bile secretion was also affected (Figure 6F). We found that LRPF reduced iron deficiency-induced perniciousness by regulating steroid hormone biosynthesis (Figure 6G) and bile secretion (Figure 6H).

## 3. Conclusions

In this study, LRPF was synthesized and studied in detail. The synthesis of LRPF was successfully optimized by using RSM. The optimum conditions were pH 9, a mass ratio of trisodium citrate to LRP of 0.45, and a reaction time of 3 h. Under the optimal conditions, the resulting LRPF is a reddish-brown powder, and the iron content of LRPF reaches 21.53%, which indicates that the model is satisfactory and accurate. In antioxidant studies, LRPF has significant antioxidant effects both in vitro and in vivo, and has strong antioxidant activity. In addition, we also revealed the mechanism of iron deficiency on the metabolic process of organisms from the perspective of gut microorganisms and blood metabolism. Through animal experiments, LRPF has been shown to be effective in treating iron deficiency anemia and significantly ameliorates the negative effects of iron deficiency by regulating the abundance of gut microbes and steroid hormone synthesis. This indicates that LRPF can be used as a nutritional supplement to prevent and treat iron deficiency anemia, and can significantly improve antioxidant activity in vivo, which has great potential for development in the food and pharmaceutical fields.

## 4. Materials and Methods

### 4.1. Materials and Chemicals

The sample of *Lotus root* was purchased from the Hubei Province of China. 2,2-Azino-bis (3-ethylbenzothiazoline-6-sulphonic acid) diammonium salt (ABTS) was purchased from Sigma Chemical Co. (St. Louis, MO, USA). All other analytical grade chemical reagents were purchased from the Beijing Chemical Reagent Factory, China. The Lotus root polysaccharide (LRP) was extracted from lotus root powder by hot water extraction [26]. The structure of the LRP1 was examined by ultraviolet (UV) spectroscopy scan, Fourier transform infrared spectroscopy (FTIR) and nuclear magnetic resonance (NMR) (^1^H and ^13^C), which has been published [27].

### 4.2. Preparation of the LRP-Iron (III) Complex (LRPF)

The response surface methodology (RSM) was used to investigate the effects of the three variables. The levels and code of variables used in the Box–Behnken design (BBD) are shown in Supplemental Table S1. Appropriate amounts of trisodium citrate, distilled water and LRP1 were fully dissolved in the round-bottom flask, where the mass ratio of trisodium citrate to LRPF1 was between 0.25 and 0.75. 2 mol/L ferric chloride solution and 1 mol/L sodium hydroxide solution was added drop by drop to maintain the pH of the reaction solution at 7–8. The addition of ferric chloride solution was terminated when there was a brown precipitate in the solution. The supernatant and four times its volume of 95% alcohol were poured into a conical flask and mixed well. Then, it was allowed to stand for 12 h at 4 °C. Finally, centrifugation and acetone and ether were used to remove impurities, and the residue was dried in a vacuum for 12 h to obtain the sample. The iron content of the piece was detected by o-phenanthroline colorimetry [22].

After a single-factor experiment of LRPF determined the preliminary range of synthesis conditions, the synthesis process was statistically optimized by three-level and three-variable Box–Behnken design. The three independent variables selected were the pH (a) of the reaction solution, the mass ratio of trisodium citrate to LRP1 (b), and the reaction temperature (c). The iron content of LRPF was taken as the response variable. In the Supplementary Table S1, these three factors are divided into three levels. The model evaluates the impact of each independent variable on the response. Experimental design and prediction data are analyzed on Design-Expert software to estimate the response of independent variables [28].

### 4.3. Quantification of Iron Content

According to the method, we modified the detection method of the iron content [28]. Under acidic conditions, ferric iron ions are reduced to divalent iron ions by ascorbic acid. Then, phenanthroline reacted with divalent iron to form a chelate, and its absorbance was measured by UV spectrophotometer. In addition, the iron content of the sample can be calculated from the standard curve. Next, 1 mL LRPF solution (10 mg/mL), 2.5 mL 10% ascorbic acid solution, 5 mL 0.1% phenanthroline solution, and an appropriate amount of ultrapure water were poured into a volumetric flask to a volume of 50 mL. The iron content in the LRPF was calculated by measuring absorbance at 510 nm at 37 °C in a water bath for 3 h. The standard solution of ferrous ammonium sulfate in distilled water was used as a control, and the calibration curve was obtained: Y = 0.1966 − 0.0121. The range of iron detection is 0–15.32 μg/mL.

### 4.4. Physicochemical Characterization of LRPF

#### 4.4.1. UV Analysis

LRP1 and LRPF powders were dissolved in 50 mL of ultrapure water to prepare a 10 mg/mL aqueous solution. The absorbance is measured by using ultraviolet spectroscopy in the wavelength range of 200–600 nm.

#### 4.4.2. FI-IR Analysis

Note that 1–3 mg LRP1 and LRPF powders were added to 100 mg of potassium bromide (spectrally pure) and mixed separately, ground evenly, and then the sample was placed in the mold under pressure (5–10 t/cm^2^) for about 5 min to obtain a transparent circular sheet. Finally, a Fourier transform infrared spectrometer was used to scan the samples in the wave-number range of 4000–400 cm^−1^.

#### 4.4.3. Thermogravimetric Analysis (TGA)

The LRPF powder is placed on the sample plate of the thermogravimetric analyzer. The thermal stability of LRPF was detected by a thermogravimetric analyzer. Under the protection of 99.99% nitrogen, the flow was set to 30 mL/min, the detection temperature was 50–650 °C, and the heating rate was 10 °C/min.

#### 4.4.4. X-ray Diffraction Analysis (XRD)

Note that 30 mg LRP1 and LRPF samples were ground evenly in an agate mortar. After the sample plate is wiped and dried with absolute ethanol, the powdered sample is poured onto the concave surface of the sample plate. The sample loading plate was placed into the X-ray diffractometer, the analysis procedure was set to time 0.05 s, and the modes were selected as Z and Tα. The diffraction angle is 5–90°.

#### 4.4.5. Circular Dichroism Analysis (CD)

LRP1 and LRPF were determined by a circular dichroism spectrometer at room temperature. The scanning wavelength range was 190–300 nm, the scanning speed was 50 nm/min, the sensitivity was 2 (MB/cm), and the sample concentration was 1 mg/mL.

#### 4.4.6. ^1^H Nuclear Magnetic (^1^H NMR) Analysis

Then, 15 mg of LRP1 and LRPF powder were completely dissolved in 1 mL DMSO and transferred to NMR tubes by pipette for detection. NMR ^1^H spectra were recorded with a Bruker advance iii-hd-500 MHz spectrometer (USA) at 25 °C.

### 4.5. Antioxidant Activities of the LRPF

#### 4.5.1. ABTS Radical Scavenging Activity

ABTS will change into a blue-green cationic form under oxidation, but antioxidant materials can slow down the occurrence of the reaction system [29]. According to Liang et al., the measurement method is slightly modified [30]. LRPF powder was prepared in water with LRPF solutions at concentrations of 1, 2, 4, 8 and 10 mg/mL. Then, 0.4 mL of different concentrations of LRPF solution and 1.6 mL of 7 mmol/L laboratory solution were poured into a conical flask, mixed well, and allowed to stand for 10 min at room temperature. The absorbance was measured by UV spectrophotometer at 734 nm and recorded as A1. The sample solution of each different concentration was replaced by distilled water, and the above operation was repeated; the measured absorbance was recorded as A0. Distilled water was replaced with ABTS+ reserve solution, the procedure was repeated, and the measured absorbance was recorded as A2. Clearance rate (%) = [1 − (A1 − A2)/A0] × 100%.

#### 4.5.2. Metal Ion Scavenging Activity

Lipid peroxidation is considered to be one of the important causes of human aging, and the increase of metal element concentration in the human body will accelerate the oxidation process of liposomes [31]. For determination of metal ion resistance activity, 0.4 mL LRPF solutions (1, 2, 4, 8 and 10 mg/mL), 40 μL ferrous chloride solution (5 mmol/L), 80 μL phenanthrene solution and 1080 μL distilled water were poured into a conical flask and mixed. The absorbance measured at 562 nm was recorded as A1. Each sample solution of a different concentration was replaced by distilled water, and the above procedure was repeated. The final step was to repeat the above operation and record the measured absorbance as A2. Clearance rate (%) = [1 − (A1 − A2)/A0] × 100%.

#### 4.5.3. Superoxide-Radical Scavenging Activity

Superoxide anion is active in the human body and prone to hydroxyl oxidation reactions. Its oxidation products will damage cell DNA and affect human function [32]. According to Zhu et al., the measurement method is slightly modified [33]. In addition, 0.3 mL of different concentrations of LRPF solution, 900 μL tires-hydrochloric acid buffer, 90 μL 7 mmol/L pyrogallol solution and 300 μL concentrated hydrochloric acid (10 mol/L) were poured into a conical flask and mixed. After the sample was placed at room temperature for 20 min, the absorbance was measured by UV spectrophotometer at 420 nm and recorded as A1. Different concentrations of sample solutions were replaced by distilled water, and the procedure was repeated. The measured absorbance was recorded as A0; replace the pyrogallol solution with absolute ethanol, repeat the above operation, and the measured absorbance was recorded as A2. Clearance rate (%) = [1 − (A1 − A2)/A0] × 100%.

### 4.6. Iron Release In Vitro Assay of the LRPF

We measured the change of iron release from LRPF in two dissolution media. The determination method is slightly adjusted to the previously reported literature [34]. Note that 100 mg of LRPF powder and 30 mg of FeSO_4_ powder were dissolved in 100 mL of artificial gastric juice (pH = 2.0) and 100 mL artificial intestinal juice (pH = 8.0). The solution was placed in a water bath at 37 °C for 3 h to simulate the digestion process. Every 15 min, 1 mL of solution was removed for testing. After the water bath (100 °C, 5 min) and centrifugation (4000 rpm,10 min), the enzyme in the solution to be tested was removed and the supernatant was obtained. Next, 0.5 mL of supernatant, water, 1.25 mL of 10% ascorbic acid solution and 2.5 mL of 0.1% phenanthroline solution were poured into a volumetric flask and mixed well and volumetric to 25 mL. The iron content in the solution was calculated by measuring the absorbance at 510 nm in a 37 °C water bath for 3 h.

Artificial gastric juice and artificial intestinal juice were prepared according to the reported method [35]: 2.0 g sodium chloride and 3.2 g pepsin were dissolved in 50 mL of distilled water. Then, an appropriate amount of 1 M HCl was added to the solution to achieve a pH of 2.0. Artificial gastric juice was obtained by adding distilled water to a volume of 100 mL. Then, 0.5 mL of porcine bile salt solution (25.0 g/L) and 0.4 g of trypsin were dissolved in a 99.5 mL sodium bicarbonate solution (0.1 mol), and then an appropriate amount of 1 M NaOH was added to the solution to achieve pH 8.0 to obtain the synthetic intestinal solution.

### 4.7. Determination of External Stability of LRPF

Different pH often significantly impacts the polysaccharide iron complex to study the stability of LRPF at different pH levels [36]; 10 mg LRPF were dissolved into 10 mL of phosphate buffer at different pH values (2.0, 3.0, 4.0, 5.0, 6.0, 7.0, 8.0). The solution was placed at 37 °C for 4 h. Then, 1 mL of the solution is sucked into a centrifuge tube. The supernatant was obtained by centrifugation (4000 rpm, 10 min). The iron content of the samples was determined by the phenanthroline method.

Stability = residual iron content of LRPF/original iron content of LRPF × 100%

### 4.8. Animal Grouping and Treatment Protocol

All animal procedures were approved and conducted by the Animal Ethics Committee of Qingdao Agricultural University (approval No. 2019-021). Four-week-old ICR male mice were randomly divided into an iron deficiency group and a blank group. The blank group (*n* = 20) was fed with a blank diet, and the other mice (*n* = 40) were fed with a low iron diet (iron content was 12 ppm) for 4 weeks. The environmental conditions are 20–24 °C. The humidity is 40–60%, and the 12 h light/dark cycle. The mice can freely eat and drink ultrapure water during the study period. Stainless steel cages were used to separate mice to prevent iron pollution. Four weeks later, the blood was collected from the caudal vein to measure the blood-related biochemical indexes. It was measured that the hemoglobin concentration of mice in the iron deficiency group was lower than 90 g/L. It can be determined that the iron deficiency anemia model in mice has been successfully established.

Iron deficiency anemia mice were randomly put into the iron deficiency group (negative control group, FeD group), LRPF group (mice were perfused with LRPF at the dose of 20 mg/kg every day), and LRP group (mice were perfused with LRP at the dose of 20 mg/kg every day). The control group continued to be given blank feed, and the other mice were given low iron feed. After four weeks of treatment, the mice were killed, and blood and tissue samples were collected for subsequent detection and analysis.

### 4.9. Blood Test and Oxidative Stress Markers Measurement in Serum

The whole blood routine in mice was examined using ABAXIS (Union City, CA, USA). The collected blood samples were allowed to stand on ice for 30 min, then centrifuged at 3000 rpm for 10 min, and the supernatant was collected. The activities of tumor necrosis factor (TNF-α), superoxide dismutase (SOD), and catalase (CAT) in serum were detected following the instructions of the kits (SEKH-0047, BC0175, BC0205, Solarbio, Beijing, China), respectively. Glutathione catalase (GSH-Px, A005-1-2, Nanjing Jiancheng Bioengineering Institute, Nanjing, China) kits were used to check the levels of GSH-Px contents in serum according to the instructions.

### 4.10. Sequencing of Microbiota and Data Analysis

Briefly, the CTAB/SDS method was used to extract the total genome DNA in samples. Then, the V3-V4 region of the 16S rRNA gene was amplified with primers and barcodes. According to manufacturing instructions, sequencing libraries were generated with NEBNext^®^ Ultra™ IIDNA Library Prep Kit (Cat No. E7645). High-quality libraries were performed with the Illumina NovaSeq platform and PE250 model by Beijing Novogene Technology Co., Ltd. (Beijing, China). QIIME2 pipeline was adopted to carry out downstream analysis, including ASVs denoise and species annotation, and PICRUSt2 was used to predict the function [37,38].

### 4.11. Metabolite Measurements by LC-MS/MS

First, plasma samples are placed on ice for protein removal. We took an equal volume of samples from each experimental sample and mixed well as QC samples and 53% methanol in water as a blank sample. Then, LC-MS/MS analysis was performed, and the raw data was dealt with Compound Discoverer 3.1 software (Thermo Fisher Scientific, Waltham, MA, USA). Differential metabolite screening thresholds: Variable importance in the projection (VIP) > 1.0, foldchange (FC) > 1.2 or FC < 0.8 and *p*-value < 0.05. Moreover, the differential metabolites were performed via Kyoto encyclopedia of genes and genomes (KEGG) pathway analysis, and a *p*-value < 0.05 was served as a significant pathway.

### 4.12. Statistical Method

The differences between mean values were statistically tested using Student’s *t* test or one-way ANOVA followed by the Tukey test for multiple comparisons. Comparisons were considered significant at *p* < 0.05 * and *p* < 0.01 **.

## Data Availability

The data presented in this study are available in the article.

## Supplementary Materials

The following supporting information can be downloaded at: https://www.mdpi.com/article/10.3390/molecules27207106/s1, Figure S1: Effects of LRPF on blood parameters and antioxidant indexes in the model mouse of iron deficiency anemia. Table S1: Levels and code of variables used in Box-Behnken design; Table S2: Box-Behnken experimental design and the results for iron content of LRPF; Table S3: Analysis of variance of the experimental results of the BBD in extraction of LRPF.

## Sample Availability

Samples of the compounds are not available from the authors.
