# Supplementary material for: Antioxidant and Biological Activities of the Lotus Root Polysaccharide-Iron (III) Complex
Source: Molecules. 2022 Oct 21;27(20):7106. doi: 10.3390/molecules27207106 (PMC9611182; doi:10.3390/molecules27207106)
Supplement: Supplementary file 1 [file molecules-27-07106-s001.zip › molecules-1949264-supplementary.pdf]

Supplemental Table S1: Levels and code of variables used in Box-Behnken design.

Variable	Symbols	Coded levels		
	Coded	-1	0	1
PH	X_1	8	9	10
m (trisodium citrate): m (LRP)	X_2	0.25	0.5	0.75
Exaction time (h)	X_3	2	3	4

Supplemental Table S2: Box-Behnken experimental design and the results for iron content of LRPF

Run	Level				
	X_1	X_2	X_3	Absorbance	Iron content（%）
1	9.00	0.75	4.00	0.566	22.97
2	8.00	0.50	4.00	0.498	20.27
3	9.00	0.50	3.00	1.198	48.08
4	9.00	0.50	3.00	1.221	49.00
5	9.00	0.25	2.00	0.797	32.15
6	9.00	0.75	2.00	0.414	16.93
7	10.00	0.25	3.00	0.508	20.66
8	10.00	0.50	2.00	0.571	23.17
9	9.00	0.50	3.00	1.179	47.33
10	8.00	0.50	2.00	0.511	20.78
11	8.00	0.75	3.00	0.302	12.48
12	10.00	0.75	3.00	0.538	21.85
13	9.00	0.25	4.00	0.724	29.25
14	9.00	0.50	3.00	1.211	48.60
15	9.00	0.50	3.00	1.081	43.43
16	8.00	0.25	3.00	0.734	29.65
17	10.00	0.50	4.00	0.505	20.55

Supplemental Table S3: Analysis of variance of the experimental results of the BBD in extraction of LRPF

Varibles	Sum of squares	df	Mean square	F-value	p-Value
model	1.57	9	0.17	65.53	<0.0001
X_1	7.411E-004	1	7.411E-004	0.28	0.6146
X_2	0.11	1	0.11	41.63	0.0003
X_3	0.000	1	0.000	0.000	1.0000
X_1X_2	0.053	1	0.053	19.99	0.0029
X_1X_3	7.023E-004	1	7.023E-004	0.26	0.6238
X_2X_3	0.013	1	0.013	4.74	0.0659
X_1^2	0.61	1	0.61	228.62	<0.0001
X_2^2	0.32	1	0.32	120.78	<0.0001
X_3^2	0.32	1	0.32	120.13	<0.0001
Residual	0.019	7	2.670E-003		
Lack of Fit	5.942E-003	3	1.981E-003	0.62	0.6373
Pure Error	0.013	4	3.187E-003		
Cor Total	1.59	16			
R^2=0.9883	R^2_{Adj}=0.9732	R^2_{pred}=0.9278	Adeq Precisior=22.722		

Supplemental Figure S1

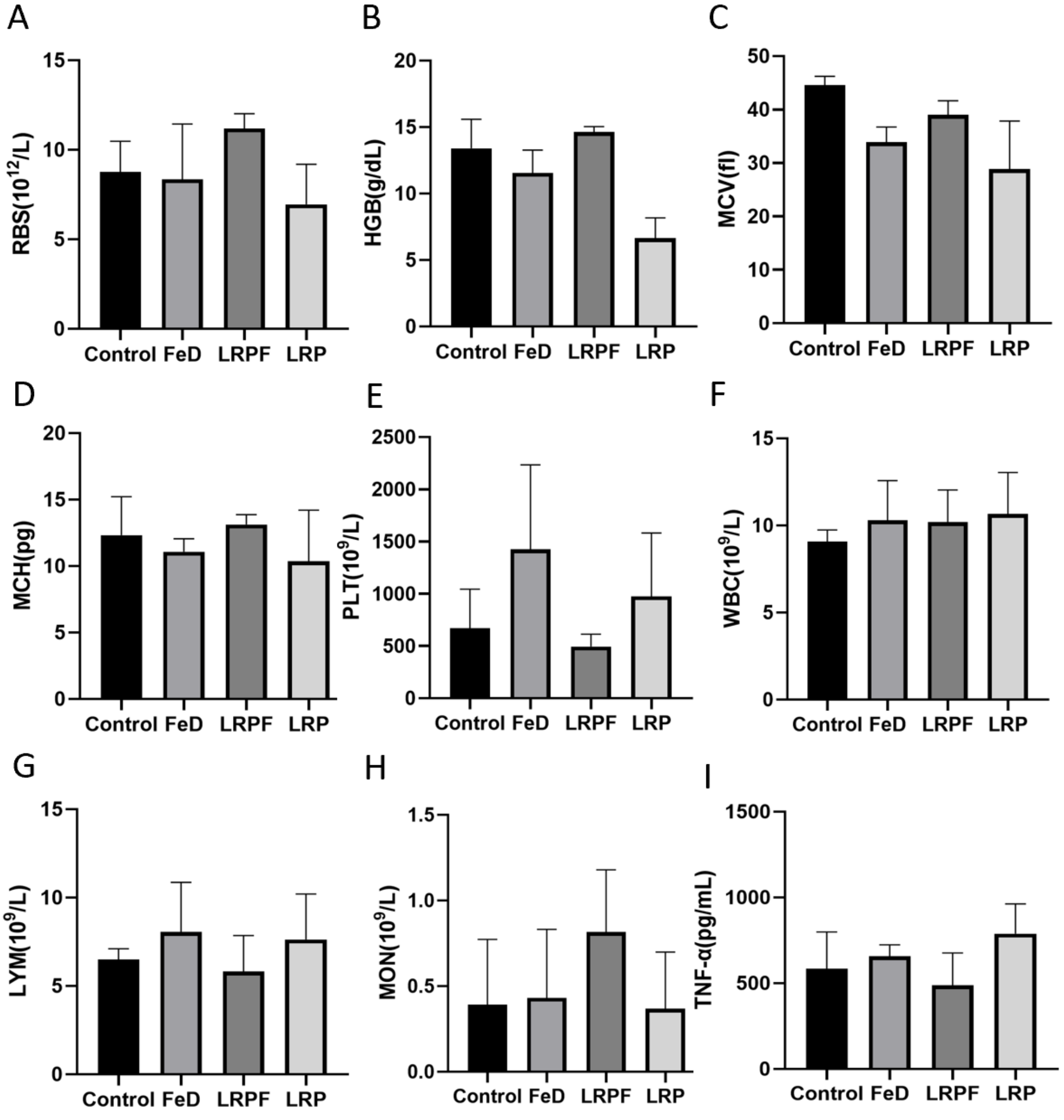

Supplemental Figure S1: Effects of LRPF on blood parameters and antioxidant indexes in the model mouse of iron deficiency anemia. (*$p<0.05$).
